# Spinal muscular atrophy phenotype is ameliorated in human motor neurons by SMN increase via different novel RNA therapeutic approaches

**DOI:** 10.1038/srep11746

**Published:** 2015-06-30

**Authors:** Monica Nizzardo, Chiara Simone, Sara Dametti, Sabrina Salani, Gianna Ulzi, Serena Pagliarani, Federica Rizzo, Emanuele Frattini, Franco Pagani, Nereo Bresolin, Giacomo Comi, Stefania Corti

**Affiliations:** 1 Dino Ferrari Centre, Neuroscience Section, Department of Pathophysiology and Transplantation (DEPT), University of Milan, Neurology Unit, IRCCS Foundation Ca’ Granda Ospedale Maggiore Policlinico, Milan, Italy; 2 Human Molecular Genetics, International Centre for Genetic Engineering and Biotechnology, Trieste, Italy

## Abstract

Spinal muscular atrophy (SMA) is a primary genetic cause of infant mortality due to mutations in the Survival Motor Neuron (*SMN*) 1 gene. No cure is available. Antisense oligonucleotides (ASOs) aimed at increasing SMN levels from the paralogous *SMN2* gene represent a possible therapeutic strategy. Here, we tested in SMA human induced pluripotent stem cells (iPSCs) and iPSC-differentiated motor neurons, three different RNA approaches based on morpholino antisense targeting of the ISSN-1, exon-specific U1 small nuclear RNA (ExSpeU1), and Transcription Activator-Like Effector-Transcription Factor (TALE-TF). All strategies act modulating *SMN2* RNA: ASO affects exon 7 splicing, TALE-TF increase *SMN2* RNA acting on the promoter, while ExSpeU1 improves pre-mRNA processing. These approaches induced up-regulation of full-length SMN mRNA and differentially affected the Delta-7 isoform: ASO reduced this isoform, while ExSpeU1 and TALE-TF increased it. All approaches upregulate the SMN protein and significantly improve the *in vitro* SMA motor neurons survival. Thus, these findings demonstrate that therapeutic tools that act on *SMN2* RNA are able to rescue the SMA disease phenotype. Our data confirm the feasibility of SMA iPSCs as *in vitro* disease models and we propose novel RNA approaches as potential therapeutic strategies for treating SMA and other genetic neurological disorders.

Spinal muscular atrophy (SMA) is an autosomal recessive neuromuscular disorder characterized by lower alpha motor neuron (MN) degeneration, which results in progressive muscle weakness, paralysis, and often death^1^. SMA is the primary genetic cause of infantile mortality, with an estimated incidence of 1 in 6,000 to 10,000 live births and a carrier frequency of 1:40 to 1:60^2^. The spectrum of SMA disease severity comprises four clinical subtypes (SMA types I-IV) based on the age of onset, highest motor function achieved, and age at death^3^.

SMA is caused by mutations in the *Survival Motor Neuron 1* (*SMN1*) gene on chromosome 5q, which encodes the ubiquitously expressed SMN protein^4^. SMN is involved in the biogenesis of small nuclear ribonucleoproteins (snRNPs) and in mRNA splicing. However, the exact role of SMN protein in the pathogenesis of SMA and the reasons for the selective loss of MNs remain largely unknown^5^.

On the same chromosome, the human genome harbors a second, almost identical copy of *SMN1*, termed *SMN2*. The two genes differ by only a few nucleotides, one of which is a single-nucleotide, C-to-T transition that leads to the alternative splicing and exclusion of exon 7 from the *SMN2* transcript^6^. The majority of the expressed protein is unstable and non-functional; it is rapidly degraded by cellular enzymatic activity owing to inefficient oligomerization^7^. However, 15% of SMN protein derived from *SMN2* is a full-length functional protein that provides partial disease modification^8^. Indeed, an inverse correlation is observed between *SMN2* copy number and the severity of the disease phenotype^9^. Several *SMN2*-targeted therapeutic strategies have been developed that aim to upregulate SMN protein expression^3^. Antisense oligonucleotides, including phosphothiorate oligonucleotides or morpholino oligonucleotides (MOs), can effectively modify the RNA splicing of *SMN2*, providing a possible therapeutic strategy^3^. We and other groups have demonstrated that oligonucleotides targeting the ISS-N1 region of *SMN2* are able to effectively upregulate SMN in *in vivo* SMA rodent models, rescuing their phenotype^6^,^10^,^11^,^12^. Phase III clinical trials with phophothiorate-modified oligonucleotides sponsored by ISIS Pharmaceutical are ongoing in SMA patients (www.clinicaltrial.gov).

The SMA phenotype can be reproduced to some extent *in vivo* in several transgenic mouse models that have been made available over the years. However, given the fundamental biological differences of the murine genome, including the lack of *SMN2*, there is no equivalent animal model that is a perfect match for the human disease. This situation represents a fundamental limit regarding the translation of preclinical therapeutic studies into human clinical trials. The possibility of generating iPSCs from human adult fibroblasts has provided unprecedented opportunities for modeling neurological diseases, including SMA^13^. In our previous work, we generated iPSC lines derived from two individuals affected by SMA type I, and observed the reduced survival of iPSC-derived MNs in long-term culture^14^. Data from our experiments also proved to be consistent with the work of other groups^13^,^15^,^16^.

In the present study, we expanded the number of our SMA cell lines by generating novel iPSCs with viral and non-viral protocols in order to test the reproducibility of our previous experiment. Indeed, we demonstrated that cell survival in long-term culture is universally reduced in MNs derived from all iPSC lines. Furthermore, we showed that the disease phenotype observed in SMA MN cultures was rescued by the increase of SMN via oligonucleotides with morpholino (MO) chemistry targeting the ISS-N1 region of *SMN2*. We also tested the ability of an exon-specific U1 small nuclear RNA (ExSpeU1s) to rescue *SMN2* pre-mRNA splicing. ExSpe U1s are modified U1 snRNAs that interacting with intronic sequences downstream of the 5′ splice site (ss) through complementarity, improves exon skipping caused by different types of mutation. This method was recently proven to correct splicing defects in different human diseases^17^,^18^. Here, we demonstrated that ExSpe U1 promoted significant correction of endogenous SMN2 Exon 7 splicing and resulted in the restoration of the corresponding SMN protein levels in SMA iPSCs and iPSC MNs.

Transcription activator-like effector (TALE) proteins are emerging as a novel tool for targeting specific DNA sequences in the field of transcriptional modulation and genome editing^19^. In particular, TALEs fused to transcription factors (TALE-TFs) may be employed to specifically induce the expression of a gene of interest^19^. Here, for the first time, we successfully tested the potential therapeutic use of TALE-TFs targeting the *SMN2* promoter with the aim of inducing the expression of the endogenous *SMN2* gene at a therapeutic level.

## Results

### Generation of iPSC lines from SMA patients

**Figure 1:**
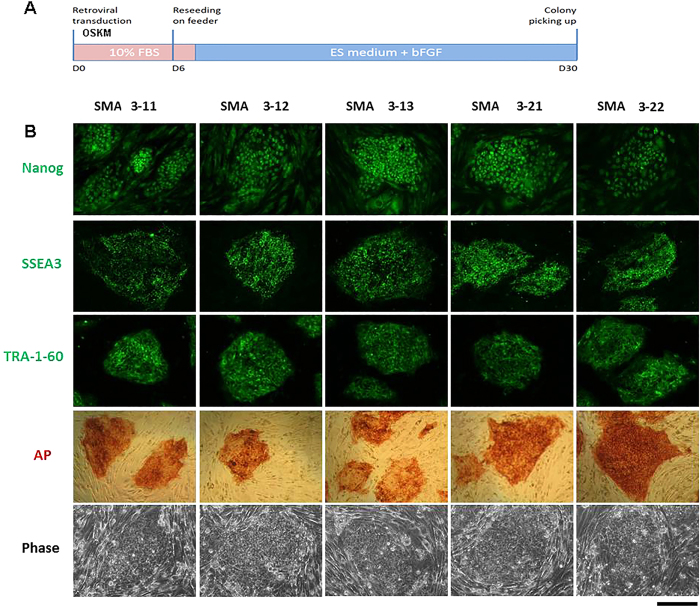
Generation and characterization of SMA iPSC lines. **(A)** Scheme of the iPSC generation protocol. Human iPSCs were generated by transducing human fibroblasts with retroviruses that encoded four reprogramming transcription factors (*OCT4, SOX2, KLF4,* and c-*MYC*). (**B)** When cultured under standard human ESC culture conditions, the morphology of human iPSCs is identical to that of human ESCs. The cells also express the pluripotency markers NANOG, SSEA3, and TRA-60-1, and demonstrate strong endogenous alkaline phosphatase activity (AP). Contrast phase images show the typical ESC morphology. Scale bar: 75 μm.

In an earlier work, we generated iPSCs from fibroblasts from two patients with type 1 spinal muscular atrophy (SMA1)^14^. In order to reproduce and further characterize the SMA phenotype in other cell lines, we generated iPSC lines from two SMA patients with a viral protocol (using retrovirus)^20^ and with a non-viral method from one SMA patient as described previously^14^ (Fig. 1A, Fig. S1, and Table 1).

Regarding the viral protocol, human iPSCs were generated with the classical Yamanaka protocol^20^ by transducing human fibroblasts with retroviruses encoding the four transcription factors (*OCT4, SOX2, KLF4,* and c-*MYC*) that have been demonstrated to reprogram somatic cells to pluripotency. The cells were isolated using morphological selection criteria without the use of fluorescent marker or drug selection. In human pluripotent stem cell culture conditions, the morphology of human iPSCs is similar to that of human ESCs. The cells (>3 clones per patient) express the pluripotency markers NANOG, SSEA3, and TRA-60-1, and show strong endogenous alkaline phosphatase activity (Fig. 1B).

We also generated cell lines from a third patient through a single nucleofection with episomally expressed oriP/EBNA1-based pluripotency factors (*OCT4, SOX2, NANOG, LIN28, c-MYC,* and *KLF4*), as described^14^, (Fig. S1A, B). Clones were generated and characterized for pluripotency markers, including expression of cell surface (SSEA3, SSEA4, TRA-1-60, and TRA-1-81) and nuclear (OCT4, NANOG, and SOX2) proteins (Fig. S1C). The SMA and wild-type (wt) lines used in this work are described in Table 1.

### SMN upregulation in SMA-iPSCs using MOs

**Figure 2:**
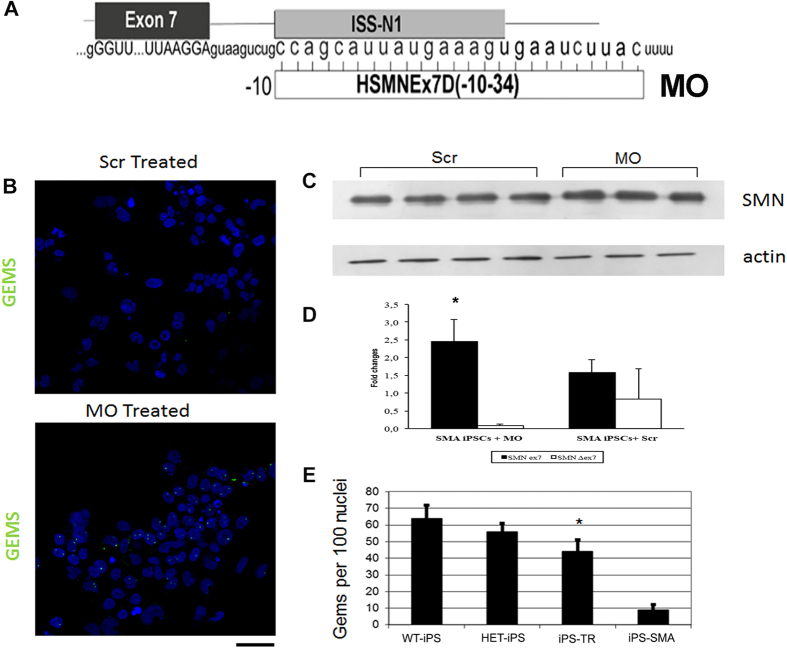
MO-10-34 increases SMN in SMA iPSCs. **(A)** Schematic representation of the PMO-10-34 sequence and its interaction with the ISS-N1 target of *SMN2* pre-mRNA. **(B)** After treating iPSCs with PMO-10-34, immunocytochemical analysis revealed a greater representation of nuclear gems (green) in MO-treated SMA iPSCs than in scrambled SMA-treated iPSCs. Nuclei are labeled with DAPI (blue). Western blot **(C)** and quantitative RT-PCR **(D)** analyses demonstrated a significant >2-fold increase in the expression of full-length SMN protein in treated SMA iPSCs compared with scrambled SMA-treated iPSCs (*p < 0.01; ANOVA). Values represent means ± s.e.m. from five independent experiments performed in triplicate. **(E)** Gems quantification confirmed the increase in the number of gems in MO-treated vs. scramble-treated cells. Gems counts in wild-type (WT) and heterozygous (HET) cells are shown for comparison (*p < 0.01, ANOVA). Scale bar: 75 μm. Values represent means ± s.e.m. from five independent experiments performed in triplicate.

Previous works by us and others have described the possibility of upregulating SMN levels by modifying *SMN2* splicing with antisense oligonucleotides (ASOs)^6^,^10^,^11^,^12^. MO is a particular type of ASO; its modified backbone imparts greater safety, stability, and efficacy, which allowed us to rescue the SMA phenotype in transgenic mice^11^. We decided to test the MO sequence (MO-10-34), already proven by us to be effective in *in vivo* SMA models, *in vitro* in SMA patient-specific iPSCs^11^. MO-10-34 is able to block intron splicing silencer (ISS-N1), and allows the inclusion of exon 7 in the *SMN2* transcript, thus converting *SMN2* into a functional *SMN1*-like gene able to produce a sufficient amount of functional SMN protein (Fig. 2A). After treating iPSCs with MO-10-34, we performed immunocytochemistry (Fig. 2B), western blotting (after 7 days) (Fig. 2C), and real time analyses (72 hours) (Fig. 2D). We observed a significant increase in the expression of SMN protein (1,6 fold increase vs. scramble-treated cells used as negative controls; P < 0.01), along with a greater representation of nuclear gems (44 ± 7 treated cells vs. 9 ± 3 scramble-treated cells used as negative controls, P < 0.01 ANOVA; Fig. 2E). Gems are specific protein-nuclear complexes that form when the SMN protein is present. They are essential for protein splicing and are absent in SMA cells. The rescue of gems in MO-treated SMA cells was remarkable.

### Treatment of spinal motor neurons with MO-10-34

**Figure 3:**
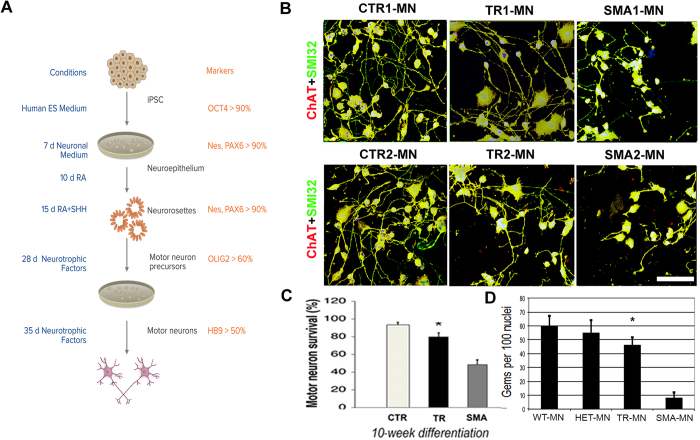
MO-10-34 ameliorates the SMA phenotype in iPSC-MNs, increasing cell survival in long-term cultures. **(A)** Schematic differentiation protocol detailing the differentiation of iPSCs into motor neurons (MNs). **(B)** iPSCs (wild-type (WT), treated (TR)-SMA iPSCs, and scramble SMA-treated iPSCs) differentiated into MNs, and expressed typical MN markers: SMI32-positive (green) and ChAT-positive (red). MNs appear yellow, representing the merging of red and green colors. Nuclei are labeled with DAPI (blue). SMA-MNs derived from scramble SMA-treated iPSCs appeared smaller and more sparse than MNs derived from WT-iPSCs. Notably, MNs derived from MO-treated iPSCs presented a rescue of cell density and size in culture, similar to MNs derived from WT iPSCs. **(C)** Quantification of MNs at week 10 after differentiation from iPSCs showed increased MN survival of TR SMA iPSC–derived MNs compared with scramble SMA iPSC–derived MNs (*p < 0.001, ANOVA). Values represent means ± s.e.m. from five independent experiments performed in triplicate. **(D)** MO-treated iPSC-derived MNs presented a significantly increased number of gems relative to MNs derived from scramble-treated SMA iPSCs. Gem counts in wild-type (WT) and heterozygous (HET) cells are shown for comparison (*p < 0.01, ANOVA). Values represent means ± s.e.m. from five independent experiments performed in triplicate. Scale bar: 75 μm.

We investigated whether an increase in SMN level using MO-10-34 could rescue the SMA phenotype in differentiated iPSC-MNs. Generation of spinal MNs from all iPSC lines was achieved using a multistage differentiation protocol previously developed for human iPSCs using RA and SHH (Fig. 3A)^14^. After 4–5 weeks under differentiation conditions, cells were generated that expressed MN-specific transcription factors, such as spinal cord progenitor markers (e.g. HB9, ISLET1, and OLIG2) and pan-neuronal markers (e.g. TuJ1, Neurofilament, and MAP2). The majority of these HB9/ISLET1-positive neurons expressed Choline Acetyl Transferase (ChAT) and were positive for the MN marker SMI-32, demonstrating a MN phenotype (Fig. 3B). The *in vitro* differentiation protocol yielded a mixed cell population that included non-MN cells. Given the limited availability of surface markers to isolate MNs and purify them further, we applied a physical strategy based on gradient centrifugation. After cells were selected using this method, immunocytochemistry analysis showed that the percentage of ChAT + SMI32 + cells was 78.2 ± 8.8% for cells derived from wt-iPSCs, 73.3 ± 6.9% for cells derived from untreated SMA-iPSCs, and 74.1 ± 6.1% for cells derived from MO-treated SMA-iPSCs. Astrocytic cells were quantified. Less than 1% of cells differentiated from iPSCs expressed the astrocyte marker GFAP. Because SMA iPSC-derived MNs exhibit reduced survival in long-term culture, we increased the cell culture time (Fig. 3B). At 10 weeks, we observed a reduction of MNs in the untreated SMA-iPSC cultures compared with wt-iPSCs with respect to cell count (P < 0.001; Fig. 3C). SMA-iPSC MNs that were treated with MO-10-34 exhibited increased survival compared with MNs derived from SMA-iPSCs-MNs treated with control vector (P < 0.001, ANOVA; Fig. 3C).

The quantification of nuclear gems demonstrated that their number was increased in MNs derived from treated versus untreated SMA-iPSCs (Fig. 3D). We concluded that cellular damage in human SMA-MN caused by the defective *SMN1* gene can be rescued by *SMN2* gene modulation using MO.

### SMN upregulation in SMA-iPSCs using ExSpeU1 targeting *SMN2*

**Figure 4:**
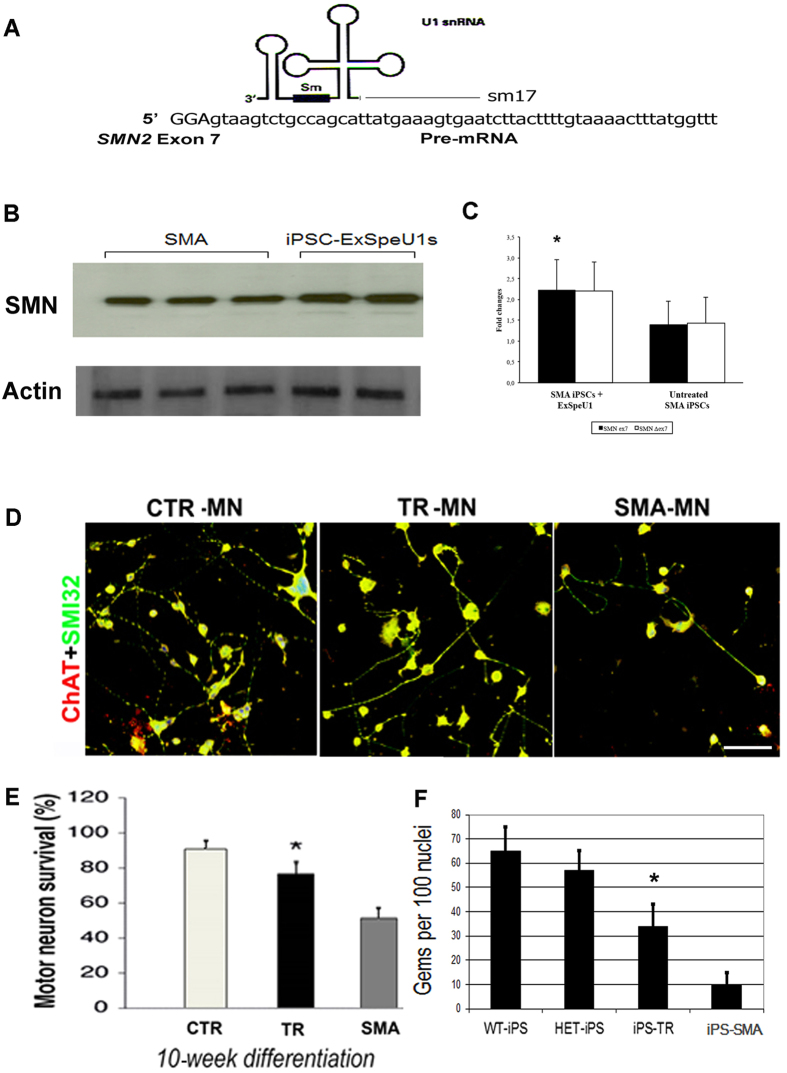
ExSpeU1 snRNA increases SMN expression in SMA iPSCs and MNs. **(A)** A schematic representation of ExSpeU1 targeting the ISS-N1 *SMN2* pre-mRNA region. **(B–C)** iPSCs nucleofected with ExSpeU1s-carrying plasmids showed a significant increase in FL SMN expression (SMN-ex7) (p < 0.01) that was detected at the protein **(B)** and mRNA **(C)** levels with Western blotting and quantitative RT-PCR analyses, respectively. **(D–F)** The motor neuron (MN) differentiation protocol was applied to wild-type (WT) iPSCs, HET iPSCs, ExSpeU1-treated SMA iPSCs, and vector control-treated SMA iPSCs. Obtained MNs expressed canonical MN markers: SMI32-positive (green) and ChAT-positive (red) cells are both expressed, and the cells appear yellow as a result of merged red and green. Nuclei are labeled with DAPI (blue). MNs derived from untreated SMA iPSC cultures appeared smaller and sparser than MNs derived from WT and HET iPSCs. Notably, MNs derived from ExSpeU1-treated SMA iPSCs presented a rescue of cell density *in vitro* compared with MNs derived from untreated SMA iPSCs **(D)**. MN quantification at week 10 of the differentiation protocol showed an increased number of ExSpeU1-treated SMA iPSC-derived MNs compared with control-treated SMA iPSC-derived MNs (*p < 0.001) **(E)**. Values represent means ± s.e.m. from five independent experiments performed in triplicate. Accordingly, MNs derived from ExSpeU1-treated SMA iPSCs presented a significant increase in gem number compared with untreated SMA iPSC-derived MNs (*p < 0.01) **(F)**. Gem counts in WT and heterozygous (HET) cells are shown for comparison. Values represent means ± s.e.m. from five independent experiments performed in triplicate. Scale bar: 75 μm.

To evaluate the therapeutic effect of ExSpeU1s on SMA-iPSCs and any corresponding SMN expression changes, we transduced SMA-iPSCs with vector containing ExSpeU1 that target the intronic sequences downstream the splice donor site of intron 7 in *SMN2* (Fig. 4A). We previously described sequence encoding ExSpeU1s for *SMN2* (sm17)^18^. After treating iPSCs with ExSpeU1, we performed immunocytochemistry, western blotting, and real-time analyses (Fig. 4B,C). We observed a significant increase in the expression of SMN protein (>1.5-fold increase; P < 0.01 vs. cells treated with control vector used as negative controls). We conclude that ExSpeU1s effectively increases SMN protein expression in SMA stem cells.

We investigated whether an increase in SMN level using ExSpeU1 could rescue the SMA phenotype in MN *in vitro*. We differentiated ExSpeU1-corrected iPSCs into MNs and enriched them with centrifugation, as described for the MO experiments. At 10 weeks, we observed more MNs in the treated SMA-iPSC-MN cultures than in the control vector-treated cells (P < 0.001) (Fig. 4D,E). We detected a greater representation of nuclear gems (34 ± 9 treated cells vs. 10 ± 5 negative control cells, P < 0.01, ANOVA) (Fig. 4F). We concluded that MN degeneration in SMA could also be rescued by *SMN2* gene modulation using ExSpeU1.

### SMN upregulation in SMA iPSCs using TALE-TFs

**Figure 5:**
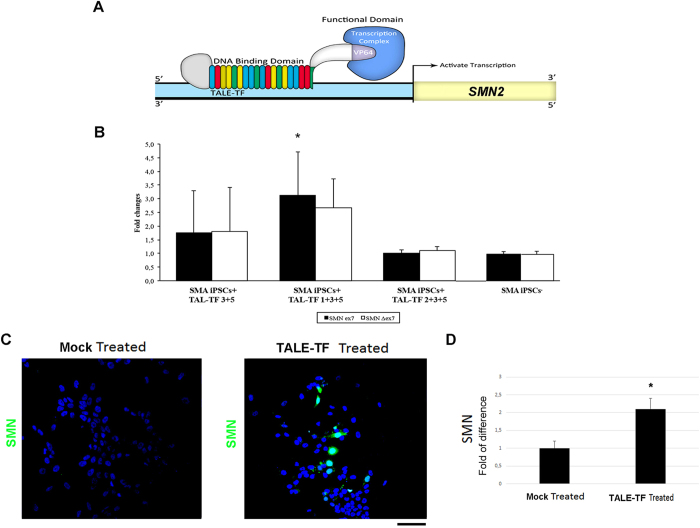
SMN upregulation in SMA iPSCs using TALE-TFs. **(A)** A schematic representation of the interaction of a TALE-TF construct with *SMN2* promoter. **(B–D)** TALE-TFs increased the transcription of the *SMN2* gene in SMA iPSCs. Quantitative RT-PCR analysis on SMA iPSCs treated with various TALE-TF combinations demonstrated the transcriptional activation of the *SMN2* gene in both the FL SMN and the SMNΔ7 transcripts, and highlighted that TALE-TF combination 1 + 3 + 5 was the most effective of all tested combinations (*p < 0.01) **(B)**. Accordingly, the immunocytochemistry assay documented a higher level of SMN protein expression (green) in SMA iPSCs transfected with TALE-TF combination 1 + 3 + 5 than in mock-transfected SMA iPSCs **(C)**. Nuclei are labeled with DAPI (blue). TALE-TF-treated cells were more brightly fluorescent than mock**-**treated cells (*p < 0.01) in quantitative analysis **(D)**. Values represent means ± s.e.m. from five independent experiments performed in triplicate. Scale bar: 75 μm.

TALE proteins are emerging as a novel tool for targeting specific DNA sequences in the field of transcriptional modulation and genome editing. In particular, TALE-TFs may be employed to specifically induce the expression of a gene of interest^19^. We tested the potential therapeutic use of TALE-TFs targeting *SMN2* promoter with the aim of inducing endogenous *SMN2* gene expression in *in vitro* models of SMA (Fig. 5A). We transfected SMA iPSCs with 12 combinations of five TALE-TF constructs (1−5, Table 2), each specifically targeting different DNA sequences within the human *SMN2* promoter. Quantitative RT-PCR analysis of SMA iPSCs transfected with TALE-TFs compared with untreated SMA iPSCs yielded results consistent with an increase in SMN expression for combinations 1 + 3 + 5 (FL SMN: >2-fold; SMNΔ7: >3.5-fold), 2 + 3 + 5 (FL SMN and SMNΔ7: >1.5-fold), 2 + 3 + 4 (SMNΔ7: ~1.5-fold) and 2 + 4 + 5 (SMNΔ7: >1.1-fold) (P < 0.01 for all). From this preliminary screen, we selected the TALE-TF combinations that had most successfully upregulated SMN (3 + 5, 1 + 3 + 5, and 2 + 3 + 5) and re-tested them in SMA iPSC lines. Quantitative RT-PCR analysis confirmed our previous results regarding the transcriptional activation of endogenous SMN2 in transfected SMA iPSCs, and showed that TALE-TFs 1 + 3 + 5 were the most effective of all TALE-TF combinations employed in our experiment: respective increases in the expression of full-length SMN and SMN lacking exon 7 (SMNΔ7 isoform) by >3-fold and >2.5-fold were observed in SMA iPSCs treated with this combination (Fig. 5B). Accordingly, immunocytochemical analysis detected greater expression of SMN in SMA iPSCs transfected with TALE-TF combination 1 + 3 + 5 than in mock-transfected SMA iPSCs (p < 0.01, ANOVA), as documented by a diffuse fluorescent signal (Fig. 5C). Fluorescence signifying the binding of anti-SMN antibodies to the targeted protein was significantly brighter in treated cells (p < 0.01, ANOVA), indicating the transcriptional activation of endogenous SMN2 (Fig. 5D). These data suggest that specific TALE-TFs targeting the *SMN2* promoter upregulated SMN expression in our *in vitro* iPSC model of SMA. Therefore, TALE-TF technology may be successfully employed to treat SMA pathology and deserves further investigation.

### Comparison of SMN full-length and Delta-7 upregulation in SMA iPSCs using the three different approaches

We demonstrated that all the three approaches induced up-regulation of full-length SMN mRNA in line with their mechanism. Here we want to compare how the various approaches differently affect the proportion of full-length and Delta-7 isoform. In fact, we demonstrated by real time RT PCR and quantitative RT-PCR analyses that the three strategies, while upregulating full-length, differentially affect the Delta-7 isoform: ASO reduced this isoform, while ExSpeU1 and TALE-TF increased it (Fig. S2).

## Discussion

Despite promising therapeutic strategies in development, SMA remains a devastating neuromuscular disease without any effective treatment. The cell type that is primarily affected in SMA is fundamentally inaccessible, and the absence of *in vivo* models that naturally develop SMA or harbor *SMN2* represents an obstacle to therapeutic developments^3^. iPSCs and their differentiated cells represent a useful *in vitro* model for the elucidation of the molecular and pathological processes involved in SMA, and for screening novel therapeutic approaches^13^.

In this study, we derived human iPSCs from SMA patients using viral and oriP/EBNA1-based transgene-free vectors, expanding our patients’ specific cell library. The use of a non-viral method allowed us to obtain iPSCs without genetic modification. The described use of SMA iPSC-derived MNs may contribute to the implementation of knowledge about the pathogenic mechanisms of SMA and the selectivity of MN loss in the context of SMN protein depletion. We observed that SMA MNs exhibit specific disease alterations secondary to the SMN defect, including a reduction in mean survival. These data were consistent with our previous results^14^. We concluded that our SMA model is able to recapitulate at least some aspects of the human disease, and may be useful for potential drug screening. Thus, we used our iPSC *in vitro* model of SMA to test novel RNA-based therapeutic strategies.

Researchers have focused on developing SMA therapies for several years. Various strategies have been investigated, mainly to directly upregulate or stabilize *SMN2* RNA and protein levels^3^. Given that *SMN2* is able to produce a small amount of full-length SMN protein through alternative splicing, one possible strategy is to target *SMN2* to increase SMN protein production. With this purpose, we applied three different molecular strategies (MOs, ExSpeU1, and TALE-TF) to induce RNA modifications in iPSCs and MNs by modifying ISS-N1, modulating the exon-intron splicing region in *SMN2* exon 7, or activating *SMN2* transcription. These three methods are specific, overcome the limits of exogenous gene introduction, and are applicable to other human genetic diseases. Here, we demonstrated that three corrective RNA approaches increase SMN protein and rescue neuropathological features of SMA in MNs.

The first approach that we tested in our iPSC model was ASO with MO chemistry. ASO technology has enabled us (and others) to restore SMN expression in SMA mice, often achieving complete rescue of disease phenotype in *in vivo* models^3^. MOs targeting *SMN2* were active and well tolerated, with impressive results in SMA mice^3^,^11^. Our current *in vitro* results confirm and extend previous data, indicating the excellent efficacy of MOs in redirecting *SMN2* splicing, thus improving the SMA disease phenotype at molecular and cellular levels in human cells. Our findings confirm that iPSCs and MNs can effectively screen oligonucleotides or other splicing approaches quickly and cost-effectively in a human-relevant model.

The most common ASO chemistries currently used to modify the splicing of *SMN2* are phosphorothioate oligonucleotides and similar variants (e.g., 2′-O-methyl phosphorothioate, 2OMePS), which have yielded encouraging results in preclinical studies and clinical^3^. A phase 3 clinical trial in SMA type I and type II-III patients with phosphorothiorate oligonucleotides is ongoing (www.clinicaltrial.gov). However, their therapeutic application can be limited because of toxicity at higher doses^21^. In contrast, the MO backbone is demonstrably effective and well tolerated, with no drug-related adverse effects in completed clinical trials in which an MO was administered systemically at a relatively high dose to boys with Duchenne Muscular Dystrophy^22^. MO stability can allow better efficacy *in vivo*^22^.

A great advantage of RNA therapy in SMA compared with other approaches (such as gene therapy) is that the *SMN2* gene remains expressed under its own endogenous promoter, subject to its regular turnover, thus obviating the need for transgene introduction. Disadvantages of potential ASO therapy for SMA in humans include the likelihood of relatively invasive delivery methods, such as continuous infusion through an implantable intrathecal or intraventricular catheter, or multi-dose bolus injection. The exact biodistribution of MO into the CNS after intrathecal injection has yet to be defined. However, this approach has an excellent safety profile, and suspending the treatment can reverse its effect.

ExSpeU1 and TALE-TF strategies to modulate *SMN2* splicing and expression represent valid alternatives to MOs and other ASOs as novel treatments for SMA. These methods can permanently correct the defect (like gene therapy) without introducing an exogenous gene, and are regulated by physiological promoter sequences (like ASOs). In fact, ExSpeU1 and TALE-TF sequences can be transferred *in vivo* with adenoviral-associated vectors (AAV) such as serotype 9, a vector that can cross the blood brain barrier and transfect the CNS safely and effectively^3^.

We also tested ExSpeU1s as a novel strategy to correct natural splicing defects in SMA, following promising results from our previous work^18^. The *SMN2* gene is a classic model of exon skipping caused by a synonymous nucleotide variant in an exonic regulatory sequence, and its modulation forms the basis for several therapeutic strategies under development for SMA, as mentioned above^3^. Nevertheless, ExSpeU1s have not been widely investigated in SMA.

Few studies have investigated the role of U1 snRNAs in the splicing correction of donor site mutations, or its potential therapeutic effect^23^. In these experiments, the modified tails of U1 snRNAs have few nucleotide modifications compared with wild-type sequences, and match the mutant donor sites exactly. Therefore, their action is limited to each mutation, and their possible binding to other normal 5′ splice sites might negatively modify the splicing of other alternatively spliced exons.

The ExSpeU1s tested in this study, which link non-conserved intronic *SMN2* sequences, do not interact directly with normal 5′ splice sites. Our results showed that ExSpeU1s targeting the intronic sequences downstream of *SMN2* exon 7 rescued correct splicing and recovered the corresponding SMN protein biosynthesis in affected MNs. The level of functional SMN obtained may therapeutically correct the SMA phenotype, making ExSpeU1s a promising strategy that must be explored in detail.

In vivo, *SMN2* splicing correction with ExSpeU1s carried by AAV9 may warrant exploration regarding classical gene replacement, because endogenous *SMN2* can be corrected permanently within the physiological gene expression provided by the chromosomal context and tissue-specific factors controlled through the regulation of transcription and pre-mRNA processing. Another advantage is that U1 snRNAs are naturally exported to the nucleus to target pre-mRNAs, ensuring more efficient delivery compared with ASOs. Furthermore, ExSpeU1s can enable permanent correction. It follows that proper vector delivery of U1 snRNAs would require few doses, possibly only one. Non-human primate data indicates that AAV9 is well distributed after intrathecal injection, compared with oligonucleotides^24^. We conclude that ExSpeU1s constitutes a useful new strategy to potentially correct splicing abnormalities linked with defective exon definition in SMA and in many other human disorders^18^.

After testing the beneficial effects of MOs and ExSpeU1s in correcting *SMN2* exon 7 splicing, we evaluated the possibility of activating *SMN2* transcription using TALE-TF technology. Direct activation of *SMN2* has great potential to facilitate the expression of full-length SMN protein in somatic cells, offering the opportunity to improve the current strategy for targeting this gene. The recent advent of engineered TALE-TF has opened new avenues for manipulating *SMN2* transcription by directly targeting its promoter, and thus has the potential to increase the full-length transcription of *SMN2* at a clinically meaningful level. Transcription activator-like (TAL) effectors are proteins produced by *Xanthomonas* bacteria when they infect plants. They enhance gene expression by binding host plant promoters. TAL effectors have been employed to generate site-specific gene-editing instruments by fusing target sequence-specific TAL effectors to nucleases (TALENs) or transcription factors (TALE-TFs). These proteins can identify and link specific target sequences and promote gene editing, including gene knockout, knock-in (with donor plasmid), and other effects.

We investigated different TALE-TFs that target a broad range of sequences in the *SMN2* promoter. The ability of TALE-TFs to interact with endogenous genomic loci is dependent on chromatin state, as well as on undefined events that regulate TALE DNA binding^19^. For these reasons, we tested several combinations of TALE-TFs for the *SMN2* genomic locus we aimed to target. Using a quantitative RT-PCR assay, we identified the highly effective TALE-TFs 1, 3, and 5, which were designed to target the human *SMN2* promoter. Employing an optimized TALE code and combinations of multiple TALE-TFs, we demonstrated that transcription of the endogenous *SMN2* gene approximately doubled in treated samples. For the first time, we demonstrated conclusively that TALE-TFs targeting the *SMN2* promoter can facilitate site-specific transcriptional modulation in SMA pathology and can robustly increase endogenous *SMN2* mRNA.

This is the first study to demonstrate that the activation of endogenous *SMN2* by multiple TALE-TFs is able to produce mature SMN protein, giving further proof that simultaneous use of engineered transcription activator factors is an efficacious strategy for controlling gene expression. The combined employment of multiple TFs for endogenous gene activation can avoid the potential off-target effect that might be produced by employing single activators^19^. TALE methodology opens a new field of research, which could be used to advance TALE proteins to treat SMA and other human diseases by inducing the expression of specific genes. TALEs are theoretically able to target any sequence and have already been deployed in many organisms with impressive success^19^. In the future, clustered regularly interspaced short palindromic repeats/Cas9 transcription factors (CRISPR9-TFs) can be used as an alternative approach to TALE-TFs, offering another powerful and precise strategy for modulating *SMN2* gene expression^25^. Therapeutic sequences from TALE-TFs and CRISPR9-TFs can be transferred *in vivo* with AAV9, as discussed regarding ExSpeU1.

In line with their known different molecular mechanism, the three approaches we have evaluated induced up-regulation of full-length SMN mRNA and protein but differentially affect the Delta-7 isoform. ASO acts mainly on splice site selection; accordingly, the increase of mRNA including exon 7 is reflected by a reduction of the shorter isoform that lacks this exon. TALE-TFs are transcriptional activators, and the resulting increased *SMN2* transcription levels do not affect splice site selection. In contrast, ExSpeU1 acting on *SMN2* pre-mRNA processing improves both splicing and the total amount of SMN transcript^17^, resulting in increases in both *SMN2*-derived isoforms. We observed this effect in cultured cells^17^ and in ExSpeU1-treated MN; it might have an additional positive effect in vivo. In fact, increased expression of the Delta-7 isoform clearly improves the SMA phenotype^26^, likely due to residual functional activity^27^. However, the practical clinical implications of this differential proportion between the full-length and delta-7 isoforms are unknown. Overall, in this study we successfully achieved *SMN2* targeting with three different RNA approaches in a meaningful experimental human model. Our findings may help to develop effective therapies for SMA and other genetic neurological diseases.

## Materials and Methods

### Viral reprogramming of human somatic cells

The Systembio-Euroclone custom service was employed for retroviral reprogramming. Retroviruses carrying *OCT4, SOX2, KLF4*, and c-*MYC* were added to human fibroblasts (at multiplicity of infection = 5) in 6-well plate. One day after infection, the viral supernatant was removed and medium added. On the same day, 1 × 10^6^ mitomycin C-treated or irradiated MEF cells were plated (pre-coated with 0.1% gelatin) and incubated overnight. Two days after the infection, the cells were trypsinized and plated in a 60 mm dish at cell densities of 3 × 10^4^ to 1 × 10^5^ cells. Two days later, medium was aspirated and replaced with hES medium (DMEM/F12 containing 20% knockout serum replacement, 2 mM glutamine, 0.1 M nonessential amino acids, 55 μM 2-mercaptoethanol, 10 ng/ml bFGF, 50 U penicillin, and 50 μg/ml streptomycin). The medium was changed daily with hES medium. Colony formation was detected after 3–4 weeks; colonies with hES-like morphology were picked manually for expansion in hES medium.

### Non-viral reprogramming of human somatic cells

Reprogramming of human skin fibroblasts with oriP/EBNA1-based episomal vectors, containing *OCT4*, *SOX2*, *NANOG*, *LIN28*, *c-MYC*, and *KLF4* cDNA^14^, was conducted by nucleofection of combinations of episomal plasmids (NHDF kit VPD-1001 with U-20 program, Amaxa, Walkersville, MD). After transfection, fibroblasts (1.0 × 10^6^ cells per nucleofection) were plated onto three 10-cm dishes with Matrigel (BD Bioscience) in fibroblast medium.

At 4 days post-transfection, we replaced the fibroblast medium with pluripotent stem cell medium (mTeSR, Stem Cell Inc.) for 8–10 days. At day 18 after transfection, first colonies with an iPSC-like morphology appeared. We stained one of the three 10-cm dishes of reprogramming culture with alkaline phosphatase (Millipore) to identify the eventual presence of human iPSC colonies. Between days 25 and 30, we passed the other two 10-cm dishes to fresh 10-cm Matrigel-covered dishes at a ratio of 1:3. We then isolated the iPSC colonies for further analysis. Efficiency of fibroblast reprogramming was approximately three to six colonies per 10^6^ fibroblast cells, in line with previous reports^14^.

### Immunohistochemistry of iPSCs and their derivatives

Cells were fixed in 4% paraformaldehyde for 10 min, permeabilized with 0.5% Tween-20 in PBS, and incubated to 0.1% Tween-20 with 10% horse serum. We exposed the cells to primary antibodies overnight and to secondary antibodies for 1 hour (Alexa Fluor, Invitrogen). We employed the following primary antibodies: SSEA-3 (1:100, R&D), SSEA-4 (1:500, DSHB), TRA1-60 (1:500, Chemicon), TRA1-81 (1:500, Chemicon), NANOG (1:500 Abcam), alpha-fetoprotein (1:500, DAKO), alpha-actinin (1:500 Sigma), alpha smooth muscle actin (1:500, Sigma), Desmin (1:100, Novocastra), TuJ1 (1:1000, Sigma), and glial fibrillary acidic protein (GFAP, 1:1000, DAKO; 1:500, Sigma). Alkaline phosphatase staining was performed^20^. For the analysis, we used a confocal LEICA LCS2 microscope.

### Differentiation of iPSCs into motor neurons

We generated spinal MNs using a multistage differentiation protocol developed for iPSCs^14^. To produce MNs, iPSCs were plated with neuronal medium [DMEM/F12 (Gibco, Invitrogen), supplemented with MEM nonessential amino acids, N2, and heparin (2 μg/mL, Sigma-Aldrich)]. After 10 days, we added retinoic acid (RA) (0.1 μM, Sigma-Aldrich) for neural caudalization. At day 17, we collected the posteriorized neuroectodermal cells. These clusters were suspended for 1 week in the same medium with RA (0.1 μM) and sonic hedgehog (SHH) (100–200 ng/mL, R&D Systems Inc.). On day 24, we supplemented the medium with other growth factors [e.g., brain-derived neurotrophic factor (BDNF), glial-derived neurotrophic factor (GDNF), and insulin-like growth factor-1 (IGF1) (10 ng/mL, Peprotech)]. MNs were enriched with gradient centrifugation^14^.

### Motor neuron phenotype analysis

Next, we analyzed the MN phenotype via immunohistochemistry^14^. The following antibodies were used: TuJ1 (1:200, Millipore), GFAP (1:300, SIGMA), OLIG2, (1:500, Santa Cruz), ISLET1 (1:200, Millipore), HB9 (1:200 Millipore), ChAT (1:200, Millipore), MAP2 (1:100; Sigma), and SMI32 (Covance, 1:500). For phenotypic analysis, the percentage of any given phenotype in a sample was obtained by averaging proportions of a specific cell type in each of 10 randomly chosen fields. Five independent experiments in triplicate were considered. Morphometric and MN count analysis were performed^14^. Results are expressed as mean ± s.e.m. for five independent experiments.

### MO sequences and cell nucleofection

The MO-10-34 sequence (GTAAGATTCACTTTCATAATGCTGG) was synthesized as bare MO^11^. The Scr-MO sequence (GTAACATTGACTTTGATATTCCTGG) was used as control (www.gene-tools.com). iPSCs or MNs were transfected with nucleofection. The cell pellet was resuspended in 100 μL Resuspension Buffer (Life Technologies). Twenty micrograms MO were added to the cells and mixed by vortexing. Each sample was transferred to a nucleofection cuvette and transfected using Neon System (Life Technologies). After nucleofection, the samples were transferred from the Neon Tip into the prepared culture plates containing pre-warmed medium. The cells were harvested at different time points after nucleofection (24, 48, and 72 hours; and 5–7 days).

### ExSpeU1 sequences and nucleofection

We have previously described vector and sequence encoding ExSpeU1s for *SMN2* (sm17) and control vectors^17^. We used both minigene and plasmids with hygromycin selection. After the centrifugation enrichment, iPSCs or MNs were transfected with nucleofection. The cell pellet was resuspended in 100 μL Resuspension Buffer (Life Technologies) per manufacturer’s direction. ExSpeU1 plasmids (5 μg) were added to the cells and mixed by vortexing. Each sample was transferred to a nucleofection cuvette and transfected using Neon System (Life Technologies). After nucleofection, the samples were transferred from the Neon Tip into the prepared culture plates containing pre-warmed medium. The cells were harvested at different time points after nucleofection (24, 48, 72 hours; and 5–7 days). We selected iPSC clones with hygromycin and cells that steadily expressed ExSpeU1 into MNs.

### TALE-TF sequences and transfection

Five TALE-TF constructs targeting the *SMN2* sequence were custom-generated by GeneCopoeia (Table 2). GeneCopoeia control vector was also employed. For plasmid transfection, ~100,000–300,000 cells/well were plated in a 6-well plate according to the manufacturer’s recommendation. On the day before transfection, we trypsinized and counted the cells. The number of cells plated per well was determined so that they reached 70–80% confluence at the time of transfection. The next day, we prepared transfection complexes of TALE-TFs using via fect transfection reagent transfection reagent according to the manufacturer’s instructions (3 μg TALE-TF plasmids). The cells were harvested at different time points after nucleofection (24, 48, 72 hours; and 5–7 days).

### RNA isolation, quantitative RT-PCR and RT-PCR

Total RNA was isolated from cells using the RNeasy Mini Kit (Qiagen) following the manufacturer’s protocol. Concentrations were detected with a Nanodrop spectrophotometer. Only samples with ratios between 1.8 and 2.0 were employed. For mRNA analyses, 1 μg of total RNA was reverse-transcribed using the Ready-To-Go kit (GE Healthcare) according to the manufacturer’s protocol. Reverse-transcribed material corresponding to 15 ng RNA was amplified with TaqMan Universal PCR Master Mix (Applied Biosystem) and the appropriate primers following the method described^28^ (600 nM each) in 7500 Real Time PCR System (Applied Biosystem). RT-PCR was carried out with a set of primer that spans the region from the exon 5–6 junction and exon 8 (forward 5′CTTCTGGACCACCAATAATTCC3′; reverse 5′AAGAGTTACCCATTCCACTTC3′). PCR products were separated on a 2% agarose gel and densitometric analysis was performed with Image J software.

### Western blot assay

Cells were washed with PBS and detached by scraping. Pellets were sonicated on ice for 10 min in buffer supplemented with protease and phosphatase inhibitor cocktail (Pierce) as described^14^. The lysates were centrifugated at 13,500 rpm for 10 min at 4 °C. Protein concentration was quantified by the Bradford assay (Pierce). Twenty micrograms of proteins were separated by 12% SDS-PAGE and electrophoretically transferred to a nitrocellulose membrane. Membranes were incubated with the antibodies against SMN (1:1.000 BD) and actin (1:1000, Sigma). Next, membranes were probed with secondary peroxidase-conjugated antibody (Invitrogen), and proteins were revealed with chemiluminescence assay (Amersham). Densitometric analysis was carried out using Image J software.

### Immunocytochemistry and gems analysis

This study was performed as previously described^14^. The cells seeded in slides were washed with PBS and fixed with 1:1 acetone:methanol for 5 min. Then they were incubated with a solution of 10%BSA, 2%Triton in PBS. After washing cells were probed with an antibody against SMN in 3% BSA for 1 hour (1:100, BD). Cells were washed with PBS and incubated for 1 hour with secondary antibodies (Alexa Fluor, Invitrogen). After further washes with PBS, the slides were mounted with DAPI (Invitrogen). Cells were analyzed using an SP2 AOBS confocal microscope. The gems number was quantified with previously reported protocol^14^. Two independent observers examined at least 100 cells in randomly selected fields from each slide, and recoded the gems number and the percent of cells with gem-positive nuclei in the different treatments.

### Statistical analysis

Stats Direct software was used for all statistical analyses. All counting data from immunocytochemical analyses and cell survival were expressed as mean values ± S.D or s.e.m. Differences between two means were analyzed using Student’s *t*-test (two-tailed), and differences among more than two means were analyzed using one- or two-way ANOVA. When ANOVA showed significant differences, pair-wise comparisons between means was performed using Tukey’s post-hoc test. In all analyses, the significance threshold was defined as p-value ≤ 0.05.

## Additional Information

**How to cite this article**: Nizzardo, M. *et al.* Spinal muscular atrophy phenotype is ameliorated in human motor neurons by SMN increase via different novel RNA therapeutic approaches. *Sci. Rep.* **5**, 11746; doi: 10.1038/srep11746 (2015).

## Supplementary Material

Supplementary Information

## Figures and Tables

**Table 1:** Characteristics of human fibroblast-derived SMA induced pluripotent stem cell (iPSC) lines and controls.

**iPSC line**	**Diagnosis**	**Mutations SMN1**	**Sex**	**Age (Months)**	**Reprogramming strategy**	**Reference**
SMA 1.1	SMA1	EX7-8DEL	Male	3	Non-viral 6 factors: OSKM+LN	Corti *et al.* 2012
SMA 2.1	SMAI	EX7-8DEL	Male	2	Non-viral 6 factors: OSKM+LN	Corti *et al.*, 2012
SMA 3.1	SMAI	EX7-8DEL	Male	3	Non-viral 6 factors: OSKM+LN	This report
SMA 4.1	SMAI	EX7-8DEL	Male	3	Viral 4 factors: OSKM	This report
SMA 5.1	SMA	EX7-8DEL	Female	3	Viral 4 factors: OSKM	This report
HET	Father of patient 1	Heterozygous EX7-8DEL	Male	N/Avail	Non-viral 6 factors: OSKM+LN	Corti *et al.* 2012
19.9	Healthy Donor	–	Male	Newborn	Non-viral 6 factors: OSKM+LN	Yu *et al.* 2009
CTR AC 1.1	Healthy Donor	–	Female	24	Non-viral 6 factors: OSKM+LN	Simone *et al.* 2014
CTR AC 1.2	Healthy Donor	–	Female	24	Non-viral 6 factors: OSKM+LN	Simone *et al.* 2014
CTR AC 13	Healthy Donor	–	Female	24	Non-viral 6 factors: OSKM+LN	Simone *et al.* 2014

N/Avail, Not available.

**Table 2:** TALE-TF sequences.

**TALE-TF target ID**	**Sequence**
TALE-TF target 1	TAAGCAACATGCCGAAACCC
TALE-TF target 2	TATAACACAGTGAAATGAAA
TALE-TF target 3	TTGAGAGAAATGAAAAATAT
TALE-TF target 4	TGTGGGAGGGCGATAACCAC
TALE-TF target 5	TCGTAGAAAGCGTGAGAAGT
