# Supplementary material for: Spinal muscular atrophy phenotype is ameliorated in human motor neurons by SMN increase via different novel RNA therapeutic approaches
Source: Sci Rep. 2015 Jun 30;5:11746. doi: 10.1038/srep11746 (PMC4485234; doi:10.1038/srep11746)
Supplement: Supplementary Information [file srep11746-s1.doc]

**Supplementary Data**

**Spinal muscular atrophy phenotype is ameliorated in human motor neurons by SMN increase via different novel RNA therapeutic approaches**

Monica Nizzardo, Chiara Simone, Sara Dametti, Sabrina Salani, Gianna Ulzi, Serena Pagliarani, Federica Rizzo, Emanuele Frattini, Franco Pagani, Nereo Bresolin, Giacomo Comi, and Stefania Corti

**
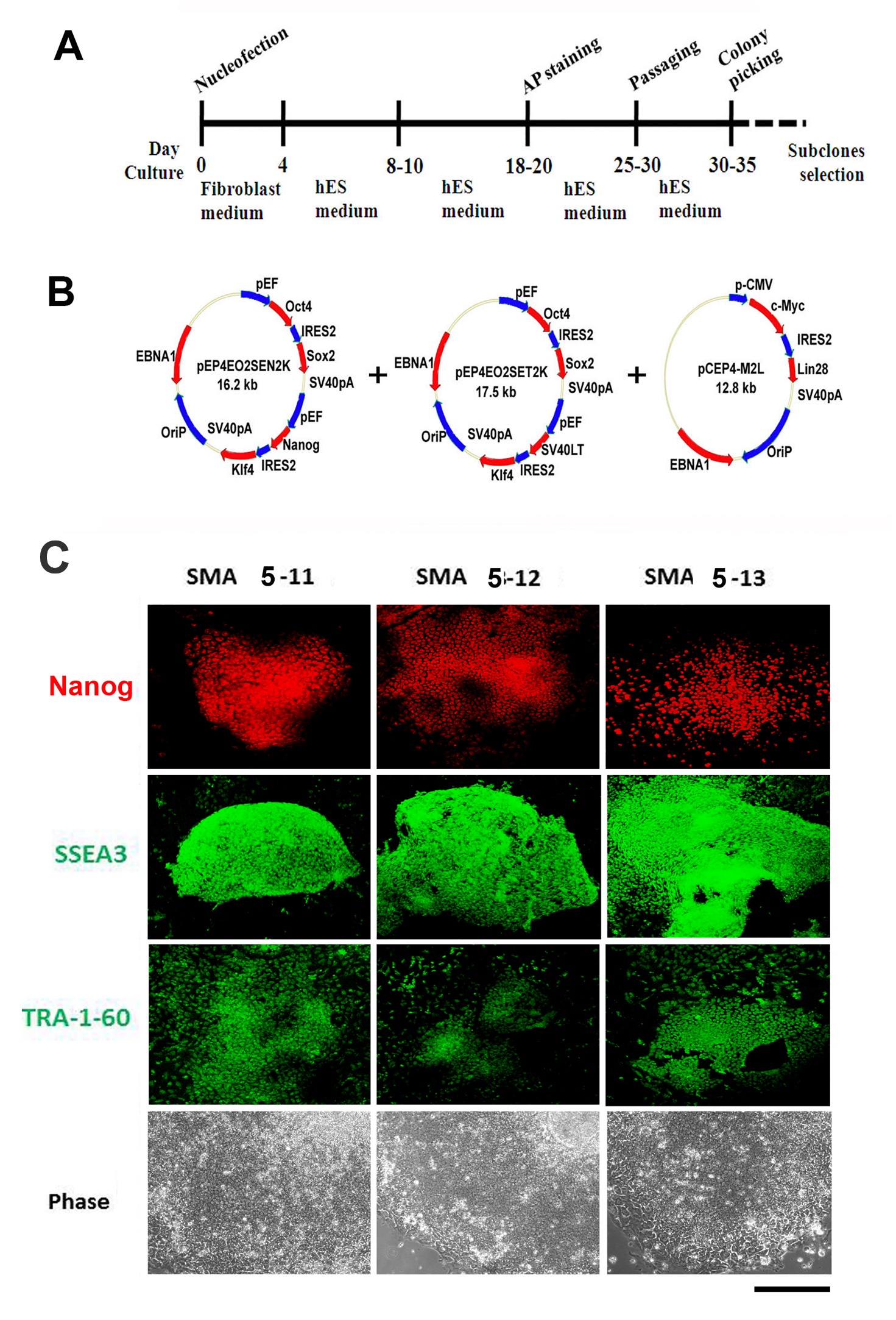
**

**Figure S1. Reprogramming of human fibroblasts without genomic vector integration, and selection of iPSC clones**

**(A)** A schematic representation of the non-viral reprogramming protocol for adult human fibroblasts. **(B)** Episomal vector maps. **(C)** Immunocytochemical analysis of iPSC clones showing the expression of pluripotency markers, including NANOG (red), SSEA-3 (green), and TRA-1-60 (red). Contrast phase demonstrated the typical iPSC colonies. Blue, DAPI nuclear stain. Scale bars: 100 µm.

**
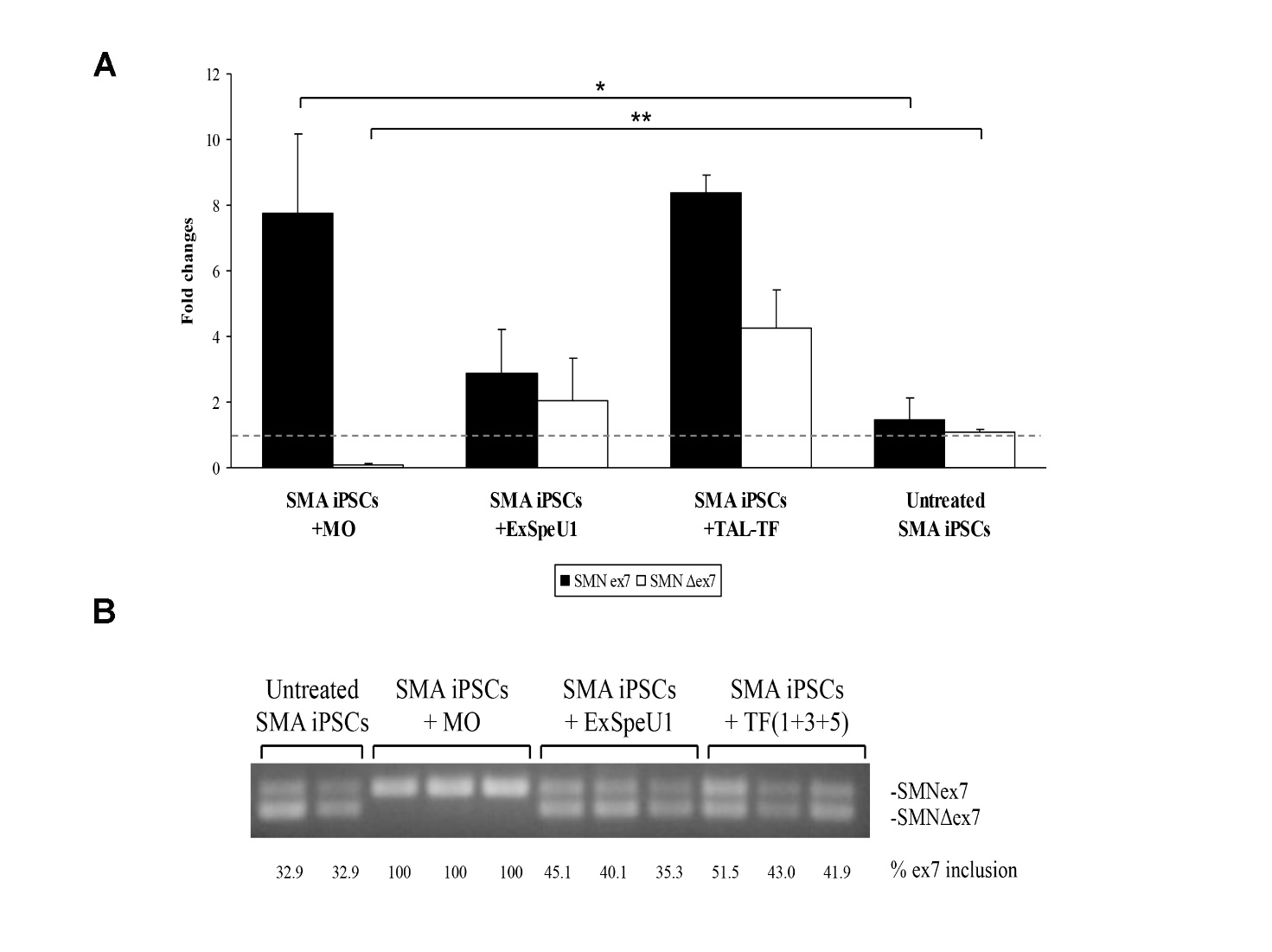
**

**Figure S2.**

**(A-B)** Real time RT PCR (A) and quantitative RT-PCR **(B)** analyses demonstrated that all the three strategies up-regulate SMN full-length transcript, but differentially affect the Delta-7 isoform: ASO significantly reduced this isoform (*P < 0.01), while ExSpeU1 (P < 0.05) and TALE-TF (P < 0.01) increased it.
